# Explore the soil factors driving soil microbial community and structure in Songnen alkaline salt degraded grassland

**DOI:** 10.3389/fpls.2023.1110685

**Published:** 2023-05-09

**Authors:** Zhenyin Bai, Aomei Jia, Haixian Li, Mingjun Wang, Shanmin Qu

**Affiliations:** ^1^ College of Animal Science and Technology, Northeast Agricultural University, Harbin, China; ^2^ College of Animal Science and Veterinary Medicine, Heilongjiang Bayi Agricultural University, Daqing, China

**Keywords:** grassland, saline-alkali degradation, bacterial diversity, fungal diversity, soil driving factors

## Abstract

**Introduction:**

Saline-alkali degradation in grassland significantly affects plant community composition and soil physical and chemical properties. However, it remains unclear whether different degradation gradients affect soil microbial community and the main soil driving factors. Therefore, it is important to elucidate the effects of saline-alkali degradation on soil microbial community and the soil factors affecting soil microbial community in order to develop effective solutions to restore the degraded grassland ecosystem.

**Methods:**

In this study, Illumina high-throughput sequencing technology was used to study the effects of different saline-alkali degradation gradients on soil microbial diversity and composition. Three different gradients were qualitatively selected, which were the light degradation gradient (LD), the moderate degradation gradient (MD) and the severe degradation gradient (SD).

**Results:**

The results showed that salt and alkali degradation decreased the diversity of soil bacterial and fungal communities, and changed the composition of bacterial and fungal communities. Different degradation gradients had different adaptability and tolerance species. With the deterioration of salinity in grassland, the relative abundance of Actinobacteriota and Chytridiomycota showed a decreasing trend. EC, pH and AP were the main drivers of soil bacterial community composition, while EC, pH and SOC were the main drivers of soil fungal community composition. Different microorganisms are affected by different soil properties. The changes of plant community and soil environment are the main factors limiting the diversity and composition of soil microbial community.

**Discussion:**

The results show that saline-alkali degradation of grassland has a negative effect on microbial biodiversity, so it is important to develop effective solutions to restore degraded grassland to maintain biodiversity and ecosystem function.

## Introduction

China has the third largest grassland area in the world, accounting for about 41% of China’s total land area (Nan, 2005; Lyu et al., 2020) and provide a range of ecological and social services, including livestock grazing (Dong et al., 2020), mitigation of global climate change (Fahad et al., 2021), soil and water conservation (Eldridge and Delgado-Baquerizo, 2017), carbon sequestration (Schlesinger and Andrews, 2000; Hu et al., 2016) and biodiversity conservation (Wang et al., 2018; Bengtsson et al., 2019). China has a total saline-alkali soil area of 35 million hectares, of which 29 million hectares are on grasslands (Pan et al., 2013). Songnen Plain, located in northeast China, is one of the three regions with the most concentrated saline-alkali soil distribution in the world (Wang and Ba, 2008). Due to overgrazing and restriction of soil parent material and hydrologic, soluble salt in deep soil is further transferred, leading to soil saline-alkali degradation in grassland (Dakouré et al., 2013; Zhang et al., 2015). Soil salinization implies changes in soil properties, such as low osmotic potential (i.e., low water availability) and ionic toxicity (i.e., high concentrations of HCO^3−^, Cl^−^, and Na^+^) of soil solutions, which inhibit plant growth, alter plant community composition, and reduce species diversity (Zhang et al., 2019; Shen et al., 2020; Gao et al., 2022). Studies have shown that grassland degradation changes soil structure, reduces the input and storage of soil organic carbon (SOC) and nutrients, and reduces soil quality (Yu et al., 2018).

Soil microbial diversity is an important basis for soil health (Zhao et al., 2019). They mediate many ecosystem functions, such as material decomposition and nitrogen sequestration, which play an important role in maintaining ecosystem stability and sustainability (Paul and Clark, 1989; Comer and Perkins, 2020). The structure and function of soil microorganisms are very sensitive to grassland salinization (Kardol and Wardle, 2010; Zhang et al., 2019). Soil salinization directly or indirectly affects soil biomes by changing vegetation composition and soil properties (Wang et al., 2018; Zhou et al., 2019). Specifically, increased salinity directly affects microbial activity by limiting soil water availability, increasing internal ion concentration, altering enzyme activity or disrupting cellular homeostasis (Van Horn et al., 2014). Studies have shown that salinity stress increases the proportion of microorganisms with “high salinity strategies” and “low salinity strategies” (Empadinhas and da Costa, 2008; Thomas et al., 2015). Soil pH, salt content and EC in alkaline soils show collinearity (Zhao et al., 2018). Different bacteria and fungi have different pH tolerance, and increased soil pH may directly affect microbial activity, diversity, and community composition (Lauber et al., 2008; Rousk et al., 2010; Singh et al., 2010; Zhang et al., 2019). In addition, grassland salin-alkali degradation also means changes in plant community composition, quantity and quality of litter and root exudates, and affects subsurface carbon and nutrient dynamics, thus altering microbial substrate (Wu et al., 2014; Li et al., 2016; Zhang et al., 2016). According to the “more individuals hypothesis”, reducing nutrient input reduces microbial abundance and thus microbial diversity (Storch et al., 2018).

Soil salinity may be an important driver of microbial community composition (Rath et al., 2019; Zhang et al., 2021). However, the extent to which salinity is a major driver of microbial community differentiation remains controversial. Zhao et al. (2019) reported that salt was a major factor affecting soil bacterial communities in abandoned salinized farmland in arid areas of China. Meta-analysis of soil microbial diversity and community has shown that microorganisms in saline soils are more affected by salinity than other environmental factors such as soil pH (Ma and Gong, 2013). In addition to soil salinity, soil pH may be another important driver of microbial community composition (Oren, 2008; Cao et al., 2021). Rath et al. (2019) reported that salinity and pH were important constraints on soil bacterial community composition. Yang et al. (2020) showed that increasing soil pH directly reduced soil total Shannon diversity, while increasing soil EC directly increased soil total Shannon diversity. However, Wang et al. (2020) showed that soil EC content in saline-alkali soil was the most important driving force for bacterial community composition in Songnen plain, rather than soil pH. In addition, studies have shown that microorganisms can use input substrates to grow even under high EC (Yan and Marschner, 2012). At present, the driving factors of microbial communities in soil with different gradients of saline-alkali degradation are still unclear. Therefore, it is of great significance to study the effects of saline-alkali degradation on soil microbial diversity and community composition for formulating reasonable grassland restoration and development programs according to different gradients of degradation.

Previous studies on the effects of different saline-alkali degradation gradients on soil microbial diversity and composition in Songnen grassland are still insufficient. Therefore, elucidating the effects of different gradients of saline-alkali degradation on soil microorganisms is of great significance for in-depth understanding of the biological ecological process and saline-alkali adaptation mechanism of saline-alkali soil, as well as mining potential microbial resources. In addition, it is crucial for reasonably developing effective solutions to restore degraded grassland ecosystem functions (Santos-Frances et al., 2019; Raiesi and Salek-Gilani, 2020). Here, Illumina high-throughput sequencing was used to analyze changes in soil microbial diversity and community composition at different saline-alkali degradation gradients in grassland. The purpose of this study was to: (1) study the effects of different saline-alkali degradation gradients on soil microbial community diversity and composition in grassland, and (2) to identify the soil factors driving soil microbial diversity and community changes in different saline-alkali degradation gradients.

## Materials and methods

### Study site

The study area is located in Fuyu County, Qiqihar City (124°0’24” E, 47°18’ 24” N, 160 m asl). Belongs to the temperate continental monsoon climate, cold in winter and warm in summer, the four seasons change obviously. Less precipitation, large evaporation. Spring has more wind, less rain drought; summer is short and hot with concentrated precipitation; autumnsunnierunny, cooling sharply, easy early frost; winter is long and cold and dry. The average annual temperature is 3.0 °C, the extreme minimum temperature is -38.5 °C, and the extreme maximum temperature is 40.7 °C. The average annual precipitation is 440.5 mm and the annual sunshine hours are 2787.1 h. The grassland belongs to warm meadow grassland. Soils are classified as castanozem, light chernozem and meadow chernozem (Wang and Ba, 2008), most of which are mixed salinity and alkalinity (pH 7 - 10.5) (Wu et al., 2021).

### Experiment design

Due to overgrazing, the grassland soils in the study area have experienced serious saline-alkali degradation. Under free grazing conditions, the spatial distribution of livestock is usually very uneven, which will lead to reduced plant coverage and increased manure in areas with more livestock, resulting in different degrees of salinization in the grassland. Therefore, in the same grassland, we qualitatively divided the grassland into three different gradients according to the salt-alkali tolerance indicator species, the relative percentage reduction rate of total grassland coverage, aboveground biomass, soil salt content (is expressed as electrical conductivity: EC) and pH, which were respectively recorded as the light degradation gradient (LD), the moderate degradation gradient (MD) and the severe degradation gradient (SD), respectively. In the light degradation gradient, the main constructive species were *Puccinellia tenuiflora* and *Chloris virgata*, with EC of 0.25 ( ± 0.02) mS/cm and pH of 8.35 ( ± 0.02). In the moderate degradation gradient, the main constructive species were *Leymus chinensis* and *Potentilla tanacetifolia*, with EC of 0.38 ( ± 0.03) mS/cm and pH of 9.01 ( ± 0.28). In the severe degradation gradient, the main constructive species were *L. chinensis* and *Carex duriuscula*, with EC of 1.29 ( ± 0.36) mS/cm and pH of 10.20 ( ± 0.03). The data of plants with different degradation gradients are shown in **Table S1**.

The soil samples were collected in July 2022. The random selection of three sampling points (5 × 5 m) on each gradient represents three replicates, and the spacing of each sampling point is 10 m. A quadrat (1 × 1 m) was arranged in each sampling point to record plant data. A five-point sampling method was used to collect topsoil soil samples (0 - 20 cm), remove plant roots in the soil, and mix five samples from the same square. A total of 9 samples (3 salinity levels × 3 replicates) were promptly packed into sterile Ziploc bags, refrigerated in ice boxes and shipped to the laboratory. The sample was divided into two parts. Part of the air drying, grinding, screening, determination of soil physical and chemical properties, and the remaining parts were stored at -80 °C for soil microbial analysis.

### Soil nutrient determination

Soil moisture content (SWC) was determined by oven drying method. In this experiment, EC was used to represent soil salt content. Soil pH and EC are measured according to (air-dried)/water (w/v 1:5) was measured with a pH meter and conductance meter (Bao, 2008). SOC was determined using K_2_CrO_7_ volume method (external heating method) (Sparks et al., 1996). Total nitrogen (TN) was determined by Kjeldahl method and alkaline hydrolysis diffusion method (Lu, 1999). The C/N ratio is calculated based on the total carbon content and the total nitrogen content of soil organic matter. The available phosphorus (AP) was extracted by 0.5 M NaHCO_3_ (pH 8.5) and determined by molybdenum blue method (UV-752 Shanghai, China) (Bao, 2008). Ca^2+^ and Mg^2+^ cations were determined by EDTA titration, and K^+^ and Na^+^ cations were determined by flame spectrophotometry. Flame photometry was used to determine exchangeable sodium (exchangeable Na^+^) in soil (ammonium acetate exchange method) (Bao, 2008).

### Soil DNA extraction, illumina sequencing and data analysis

Total DNA was extracted from 0.25 g of Soil using the FastDNA^®^ Spin Kit for Soil (MP Biomedicals, USA) isolation kit according to the manufacturer’s requirements. After DNA extraction, the extracted genomic DNA was detected by 1% agarose gel electrophoresis. For MiSeq library construction, bacterial V3 and V4 hypervariable regions were amplified using primers 338F (5’ -ACTCCTACGGGAGGCAGCA-3’) and 806R (5’ -GGACTACHVGGGTWTCTAAT-3’). Primer ITS1F was used (5’-CTTGGTCATTTAGAGGAAGTAA-3’) and ITS2 (5’-GCTGCGTTCTTCATCGATGC-3’) were used to amplify the hypervariable region of fungal ITS1. Specific primers with barcode were synthesized according to the specified sequencing region. The amplification process was pre-denaturation at 95°C for 5 min, 25 cycles of 95°C for 30 s, 50°C annealing for 30 s, 72°C for 40 s, and 72°C for 7 min of extension. PCR products of the same sample were mixed and detected by 2% agarose gel electrophoresis. PCR products were recovered by gel cutting using AxyPrepDNA gel recovery kit (AXYGEN Company) and eluted with Tris_HCl. Electrophoresis was performed on 2% agarose. PCR products were quantified with the QuantiFluor™ -ST Blue fluorescence Quantification System (Promega). The PCR products were purified with TruSeqTM DNA Sample Prep Kit to construct the Miseq library. Illumina Miseq platform was used for on-machine sequencing of the constructed library.

The reads of each sample were spliced using FLASH (version 1.2.11) software (Magoc and Salzberg, 2011), primers were removed, and sequences were trimmed to remove low-quality sequences. Sequences were clustered at 97% similarity level (OTU) and screened using USEARCH (version 10.0) with a threshold of 0.005% for all sequences (Edgar, 2013). The RDP software (Version 2.2) was then used for comparison with the Silva and UNITE databases, with confidence intervals of 80% for bacterial and fungal classification (Wang et al., 2007; Quast et al., 2013). Mothur software was used to calculate Chao1, Simpson diversity, Shannon’s evenness, and Good’s coverage to estimate bacterial and fungal diversity and richness.

### Statistical analysis

All statistical analyses were performed using R (4.1.2) software (R Core Team, 2020). Shapiro-Wilk test was used to test whether the soil physical and chemical properties, microbial α diversity and relative abundance of microbial phyla meet the normal distribution, and “log” conversion was performed on the data that did not meet the normal distribution. One-way ANOVA analysis and HSD test were used to analyze the differences of soil properties, soil microbial α diversity and the relative abundance of dominant phylum under different degradation gradients. The relationship between microbial α diversity and soil physicochemical properties was analyzed by Spearmen rank correlation analysis. Venn diagram was used to identify different degradation gradients shared and unique OTUs. Permanova was used to calculate the composition differences of bacterial and fungal communities under different degradation gradients, and Bray-Curtis distance matrix between OTUs of different samples was calculated based on the “vegdist” package (Oksanen, 2016), and similarity analysis (ANOSIM) was performed on 999 permutations. PCoA visualization of soil bacteria and fungi in different degradation gradients was performed. Linear discriminant analysis (LDA) and linear discriminant analysis (LEfSe) were used to identify the characteristics with significant bacterial and fungal differences between different degradation gradients by biomarkers, and the effect size of each characteristic was evaluated with a threshold of 3.0 and a significant α of 0.5. In order to explore the soil factors driving bacterial and fungal community composition and dominance phyla, we performed RDA analysis of soil properties and bacterial and fungal communities using the “vegdist” package. In addition, “randomForest”, “vegan” and “ggcor_master” were used to conduct randomForest and Mantel tests to investigate the soil factors driving the composition and dominance of soil bacterial and fungal communities.

## Results

### Effects of grassland degradation gradient on soil properties

To explore the factors that drive soil bacterial and fungal community diversity and composition, we investigated the physical and chemical properties of soils with different saline-alkali degradation gradients (**Table S2**). The results showed that there were significant differences in physical and chemical properties of soils with different degrees of saline-alkali degradation. Specifically, there was no significant difference in soil EC content between LD and MD (P > 0.05). Compared with LD and MD, EC content in SD soil increased by 416.00% and 239.47%. Soil pH of different gradients was significantly different (P < 0.05) and showed an increasing trend with the aggravation of saline-alkali degradation gradient. LD soil pH was 8.35 ( ± 0.15) and SD soil pH was 10.20 ( ± 0.03). SWC showed a decreasing trend as soil degradation gradient increased. Soil nutrient content (SOC, TN and AP) showed a decreasing trend with the aggravation of degradation gradient, but there was no significant difference in soil C/N among different gradients (P > 0.05). There was no significant difference in exchangeable Na^+^ content between MD and SD soils, which increased by 29.65% and 45.76% compared with LD, respectively. The contents of cations (K^+^, Ca^2+^, Mg^2+^ and Na^+^) in soil increased with the aggravation of soil saline-alkali degradation.

### Effects of grassland degradation gradient a on soil microbial community diversity and composition

After Illumina high-throughput sequencing and filtration removal of low-quality sequences, a total of 1167,100 and 1066499 effective bacterial and fungal sequences were obtained from 9 samples. The effective sequence length of bacteria was mainly distributed in 417 bp, and that of fungi was mainly distributed in 248 bp. The Good’s coverage index of bacteria and fungi in all samples were higher than 98.93% and 99.97% respectively, indicating that the sequencing quality was good.

Different degradation gradients significantly affected α diversity of bacteria and fungi (P < 0.05) (**Table 1**). The bacterial Chao1 richness decreased with the increase of degradation gradient. There was no significant difference in bacterial Chao1 richness between LD and MD, while SD significantly decreased bacterial Chao1 richness. Compared with LD and MD, the Chao1 richness in SD were decreased by 33.32% and 28.99%, respectively. For fungi, observed OTUs in SD was significantly decreased (P > 0.05). There were no significant differences in Chao1 richness and Shannon Indexes between LD and MD, while SD significantly decreased the Chao1 richness and Shannon Indexes. Compared with LD, the Chao1 richness and Shannon Indexes of SD fungi were decreased by 58.20% and 46.03%, respectively. Spearmen rank correlation analysis showed that bacterial α diversity was positively correlated with soil nutrients (SOC, TN, and AP), but negatively correlated with EC, pH, exchangeable Na^+^ and cations contents (K^+^, Ca^2+^, Mg^2+^, and Na^+^). Fungal α diversity was positively correlated with SWC and negatively correlated with exchangeable Na^+^ and Ca^2+^ contents (**Figure S1**).

**Table 1:** α diversity of soil bacteria and fungi under different degradation gradients by one-way variance analysis.

		LD	MD	SD	F	*P*
Bacteria	Observed OTUs	3317 ± 286 a	3191 ± 655 a	2271 ± 208 a	5.30	0.04
	Chao1 richness	3644.77 ± 220.80 a	3422.40 ± 631.09 ab	2430.33 ± 177.74 b	7.86	0.02
	Shannon Index	6.04 ± 0.26 a	6.25 ± 0.33 a	5.78 ± 0.40 a	1.47	0.30
Fungi	Observed OTUs	527 ± 171 ab	565 ± 146 a	220 ± 48 b	6.10	0.04
	Chao1 richness	537.71 ± 173.11 ab	575.07 ± 149.82 a	224.78 ± 46.05 b	9.12	0.02
	Shannon Index	4.28 ± 0.25 a	4.55 ± 0.35 a	2.31 ± 1.14 b	6.11	0.04

Different letters (a, b, c) indicate significant effects. LD means the light degradation gradient, MD means the moderate degradation gradient and SD means the severe degradation gradient.

**Figure 1:**
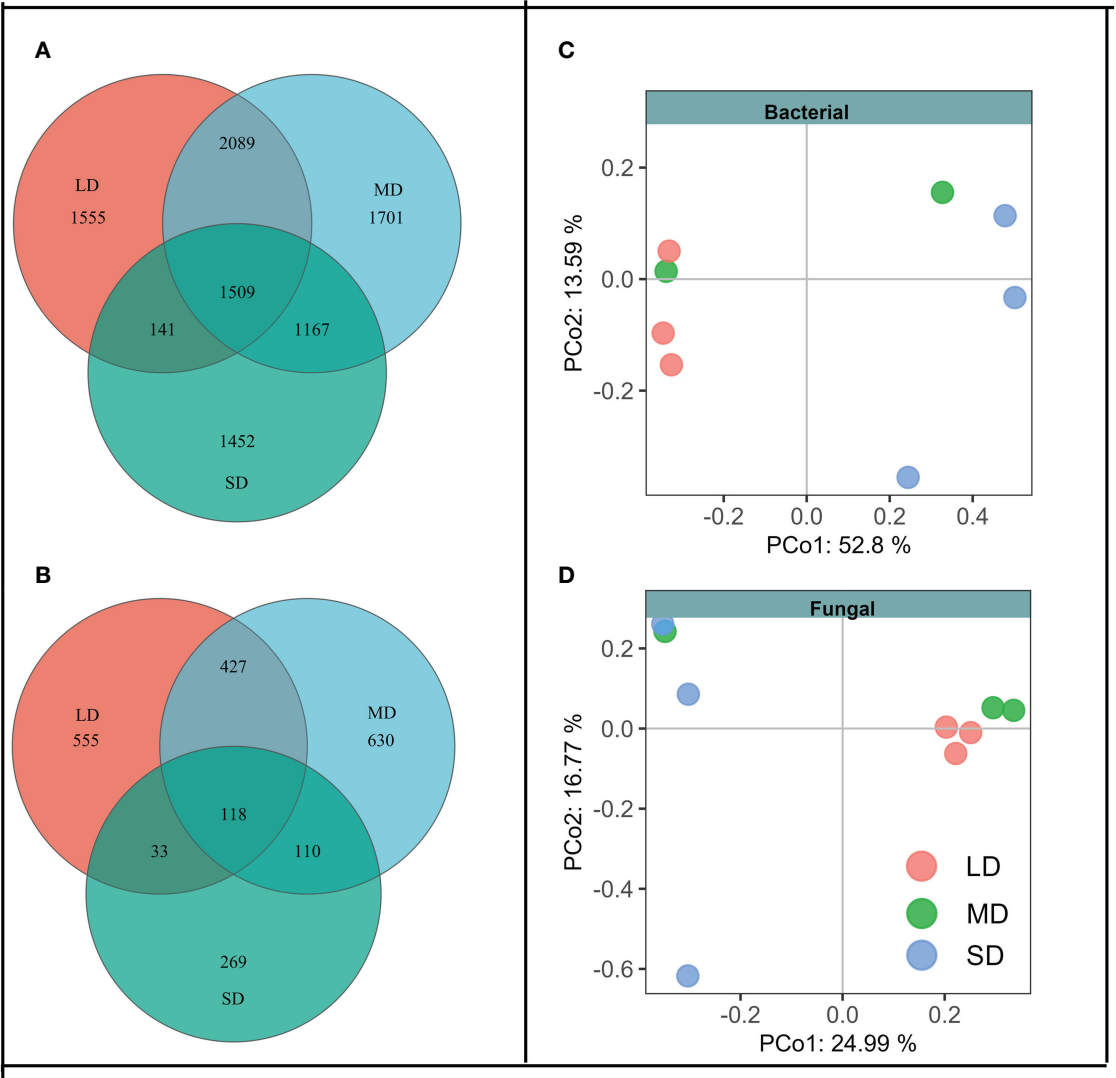
Venn diagram shows that soil bacteria **(A)** and fungi **(B)** with different degradation gradients share and have unique OTUs. Principal co-ordinates analysis (PCoA) analysis of bacterial **(C)** and fungal **(D)** community structure at different degradation gradients based on Bray-Curtis distance. LD means the light degradation gradient, MD means the moderate degradation gradient and SD means the severe degradation gradient.

The changes of soil physical and chemical properties with different saline-alkali degradation gradients make us suspect whether the changes in soil physical and chemical properties drive the changes in soil bacterial and fungal community composition. Venn diagram showed that the aggravation of saline-alkali degradation gradient changed the composition of soil bacteria and fungi community (**Figures 1A, B**). Under the same degradation gradient, OTUs unique to bacteria were higher than that of fungi. Further analysis showed that the unique bacteria OTUs belonged to phylum Sva0485 in LD, FCPU426 and CK-2C2-2 in SD, and the unique fungi OTUs belonged to Calcarisporiellomycota in MD. In addition, the Permanova analysis based on Bray-Curtis distance matrix showed that different degradation gradients had significant effects on soil bacterial and fungal communities (F = 2.95, P = 0.02; F = 1.44, P = 0.04). This result was also confirmed by PCoA, where bacterial and fungal communities were significantly separated under different saline-alkali degradation gradients (**Figures 1C, D**).

**Figure 2:**
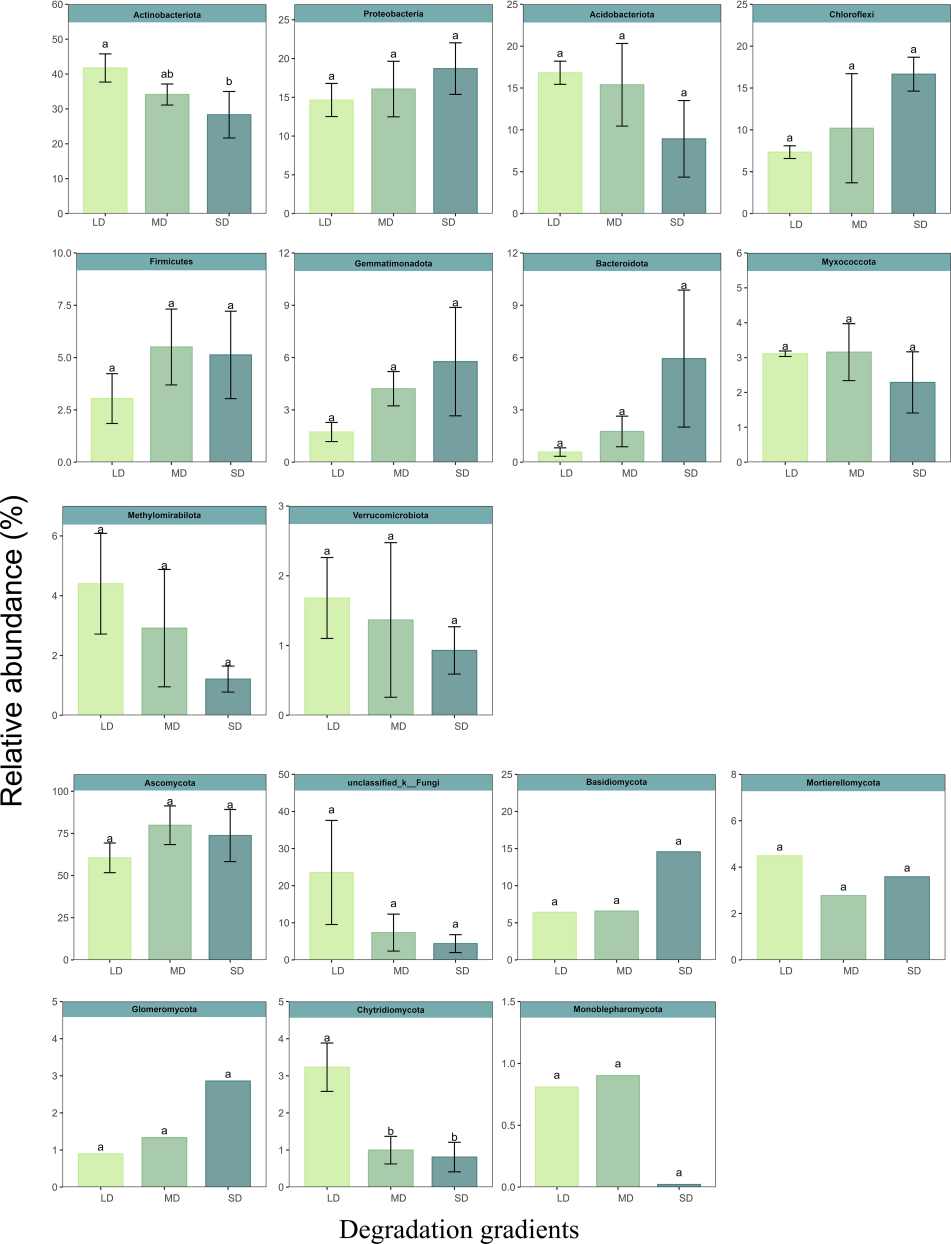
Reative abundance of dominant phylum of bacteria and fungi at different degradation stages. Different letters (a, b, c) indicate significant effects.

Under different degradation gradients, the bacterial community mainly consisted of Actinobacteriota (34.74%). Followed by Actinobacteriota (34.74%), Proteobacteria (16.47%), Acidobacteriota (13.71%), Chloroflexi (11.39%), Firmicutes (4.76%) and Gemmatimonadota (3.79%), Bacteroidota (2.76%), Myxococcota (2.85%), Methylomirabilota (2.15%) and Verrucomicrobiota (1.33%). The relative abundance of Actinobacteriota decreased with increasing degradation gradient. Compared with LD, the relative abundance of Actinobacteriota in MD and SD decreased by 18.25% and 32.12%, respectively. The fungal community was dominated by Ascomycota, and its relative abundance was greater than 71.35% at different degradation gradients. Soil saline-alkali degradation significantly decreased Chytridiomycota relative abundance. Compared with LD, the relative abundance of Chytridiomycota in MD and SD was decreased by 53.50% and 68.42%, respectively (**Figure 2**). Lefse (threshold of 3) was used to calculate the bacterial and fungal enrichment species under different grassland degradation gradients. There are six bacterial phyla (Actinobacteriota, Proteobacteria, GAL15, NB1-j, GAL15, Planctomycetota and Acidobacteriota) and two fungal phyla (Ascomycota and Basidiomycota) in LD. Actinobacteriota and Ascomycota were the most abundant phyla in MD. Eight bacterial phyla (Proteobacteria, Acidobacteriota, Chloroflexi, Bacteroidota, Myxococcota, Dependentiae, Planctomycetota and Cyanobac) were the most abundant in SD (**Figures S2A, B**).

### Relationship between soil property change and microbial composition

**Figure 3:**
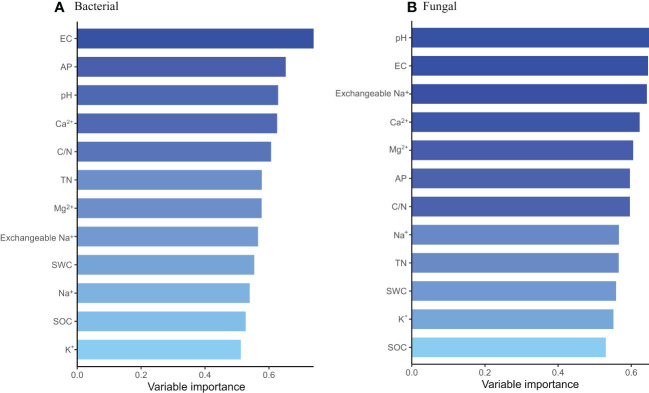
Random forest analysis of soil drivers of bacterial **(A)** and fungal **(B)** community composition at different degradation gradients.

**Figure 4:**
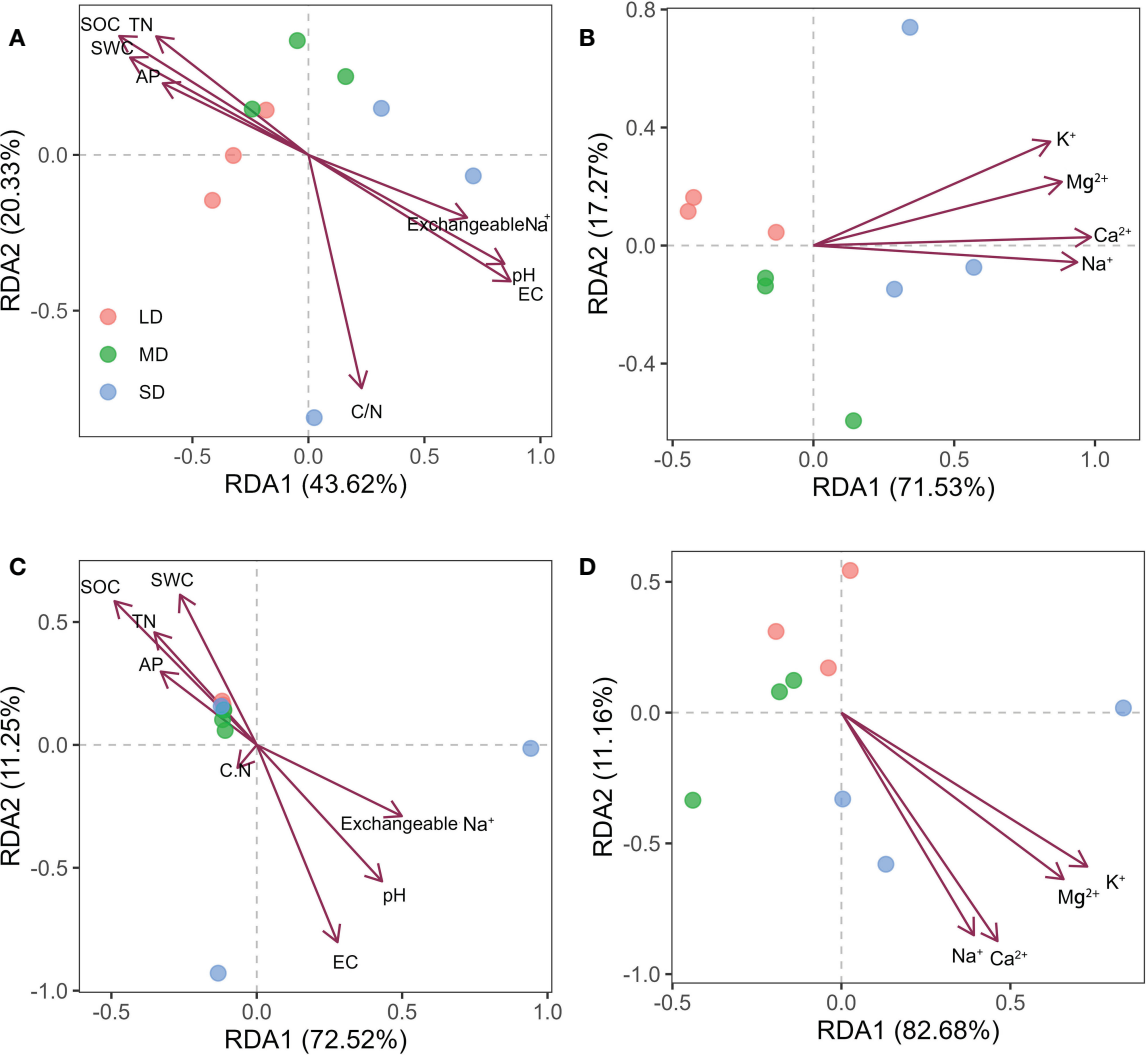
Redundancy analysis (RDA) of bacterial **(A, B)** and fungal **(C, D)** community composition and soil properties in soil with different saline-alkali degradation gradients. The arrows represent the relationship between the physical and chemical properties of the soil and the microbial community (bacteria or fungi) of the sample site.

In order to explore the soil physical and chemical properties that drive the changes of soil bacterial and fungal community composition. Random forest results showed that EC, AP and pH were the main driving factors of soil bacterial community composition, while pH, EC and exchangeable Na^+^ were the main driving factors of soil fungal community composition (P < 0.05) (**Figure 3**). Further Mantel tests showed that EC and pH were the main driving factors of soil bacterial community composition, while EC, pH and SOC were the main driving factors of soil fungal community composition (P < 0.05) (**Table 2**). RDA analysis (Redundancy analysis) also demonstrated that soil bacterial and fungal communities were negatively correlated with EC, pH, exchangeable Na^+^ and soil cations (K^+^, Ca^2+^, Mg^2+^, and Na^+^), but positively correlated with SWC and soil nutrients (SOC, TN, and AP) (**Figure 4**).

**Table 2:** Soil drivers of bacterial and fungal community composition at different degradation gradients were examined by Mental test.

	Bacterial composition		Fungal composition	
	r	P	r	P
EC	0.548	0.020	0.559	0.009
pH	0.510	0.019	0.553	0.001
SOC	0.482	0.028	0.557	0.007
TN	0.337	0.046	0.453	0.020
C/N	0.027	0.921	0.059	0.801
AP	0.231	0.197	0.302	0.102
SWC	0.173	0.329	0.258	0.155
K^+^	0.351	0.050	0.474	0.008
Ca^2+^	0.256	0.108	0.357	0.028
Mg^2+^	0.133	0.601	0.234	0.316
Na^+^	0.164	0.289	0.260	0.131
Exchangeable Na^+^	0.167	0.354	0.321	0.075

**Figure 5:**
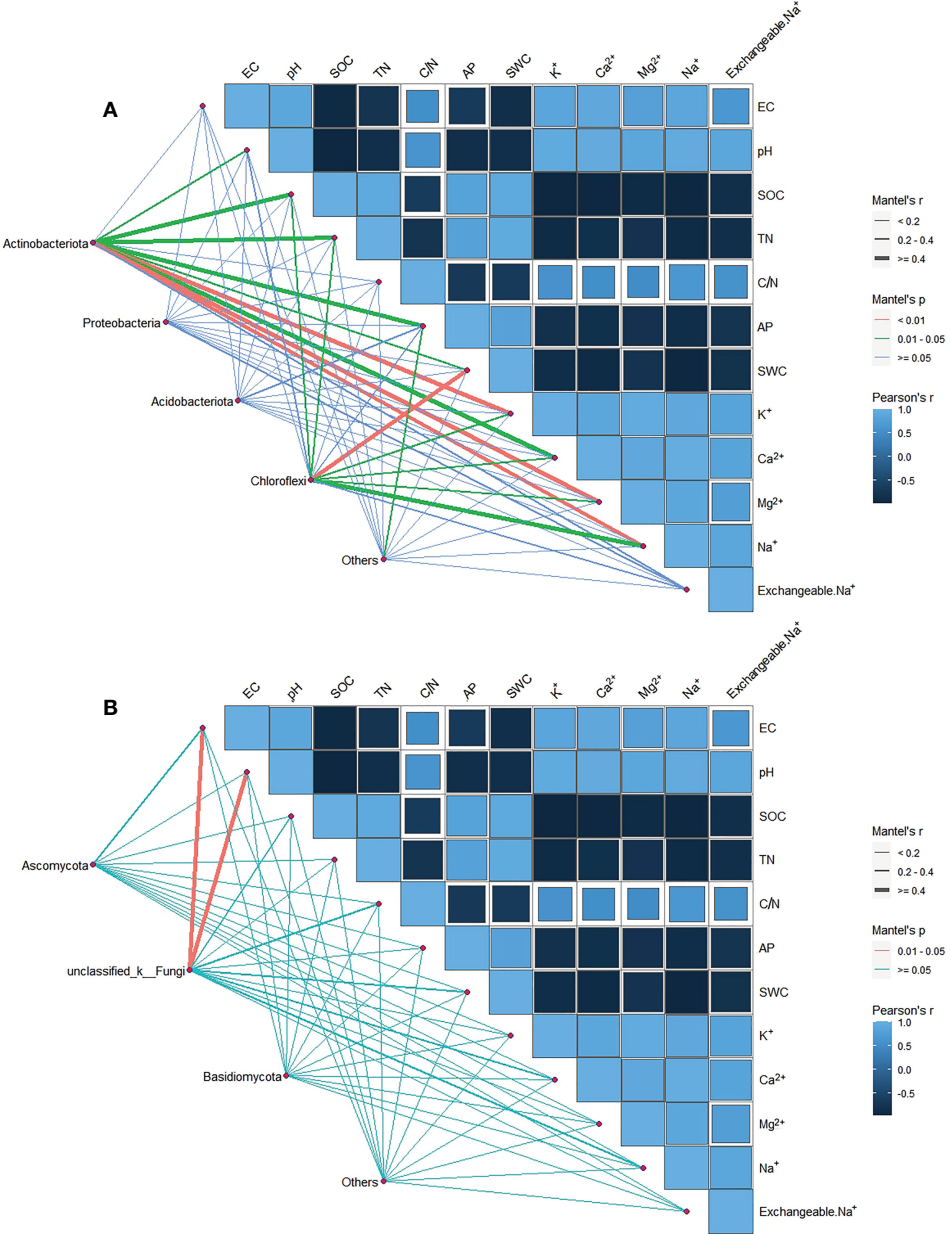
Partial Mantel test examined the relationship between dominant phyla of soil bacteria **(A)** and fungi **(B)** (relative abundance > 5%) and soil environmental factors. The edge width corresponds to the Mantel’s r statistic for the corresponding distance correlation, and the edge color indicates statistical significance.

We consider phyla with relative abundance greater than 5% as the dominant phyla. The main phylum of soil bacteria community are Actinobacteriota, Proteobacteria, Acidobacteriota and Chloroflexi, and the main phylum of soil fungi community are Ascomycota, Basidiomycota and unclassified_k_Fungi. We analyzed the correlation between species abundance and soil factors. Actinobacteriota was significantly correlated with pH, SOC, TN, AP, SWC and soil cation (P < 0.05). Chloroflexi was significantly associated with SOC, TN, SWC and soil cation (P < 0.05). Proteobacteria and Acidobacteriota had no significant relationship with soil physicochemical properties (P > 0.05) (**Figure 5A**). In terms of fungi, unclassified_k_Fungi significantly associated with EC and pH (P < 0.05), while Ascomycota, and Basidiomycota no significant relationship with soil physical and chemical properties (P > 0.05) (**Figure 5B**).

## Discuss

### Effects of saline-alkali degradation on soil microbial diversity in grassland

In terrestrial ecosystems, evapotranspiration plays a particularly important role in the salinization of arid and semi-arid lands (Yadav et al., 2011). Long-term overgrazing of Songnen grassland resulted in decreased vegetation coverage and aboveground biomass (**Table S1**), which increased soil water loss. Increased evaporation results in the accumulation of Na_2_CO_3_ and NaHCO_3_ dissolved in groundwater in the topsoil through capillary movement (Cao et al., 2021). As evaporation of Songnen grassland was significantly higher than precipitation, the accumulated salt in the soil surface could not return to the deep soil, which resulted in secondary salinization of soil. The results showed that SWC, EC, pH, SOC, TN and AP tended to decrease with the increase of soil salinization, while exchangeable Na^+^ and cations tended to increase (**Table S2**). This is consistent with the results of Gao et al. (2022), showing that saline-alkali degradation changes the physicochemical properties of soils.

Soil bacterial and fungal diversity and community composition are reported to be regulated by a variety of biological and abiotic factors, such as soil water content (Yang et al., 2019), soil pH (Zhao et al., 2018; Yang et al., 2020), Soil salinity (Rath et al., 2019; Zhang et al., 2019), SOC (Cao et al., 2021), soil nutrients (Li et al., 2016) and above-ground vegetation (Rath and Rousk, 2015). The results showed that α diversity of soil bacteria and fungi decreased with the aggravation of soil saline-alkali degradation gradient (**Table 1**). A number of studies have shown that soil bacterial and fungal diversity decreased with the increase of soil salinity (Oren, 2008; Rath and Rousk, 2015; Zhang et al., 2019). However, Wang et al. (2020) showed that α diversity of soil bacteria did not change significantly with the aggravation of grassland saline-alkali degradation. Cao et al. (2021) showed that the Shannon Index of soil fungi did not change significantly with the aggravation of saline-alkali degradation in grassland. We believe that this difference may be caused by differences in vegetation composition and soil properties. Our study further showed that bacterial α diversity was positively correlated with soil nutrients (SOC, TN, and AP) and negatively correlated with EC, pH, exchangeable Na^+^, and cations (K^+^, Ca^2+^, Mg^2+^, and Na^+^). Fungal α diversity was positively correlated with SWC and negatively correlated with exchangeable Na^+^ and Ca^2+^ contents (**Figure S1**). Salinity is widely recognized as a major determinant of microbial diversity in different habitats (Zhang et al., 2021). Salinity affects some microbiota by limiting microbial survival. At the cellular level, salinity leads to elevated internal ion concentrations, which can become toxic, for example, by altering enzyme activity or even disrupting cellular homeostasis (Wichern et al., 2006; Setia et al., 2010; Chowdhury et al., 2011; Van Horn et al., 2014). Zhang et al. (2019) showed that microbial diversity decreased linearly with the increase of salinity, and the differences between communities increased significantly with salinity. pH is another important environmental factor driving bacterial community change (Zhao et al., 2018; Yang et al., 2020), bacterial α diversity was negatively correlated with pH, possibly due to the relatively narrow growth tolerance of most bacterial groups. A decrease in aboveground plant biomass reduces litter input to soil and limits the activity and abundance of soil microbial communities (Wu et al., 2014). It was found that the decrease of aboveground biomass might be the cause of the decrease of soil C and N concentrations, which in turn decreased the bacterial Chao1 richness in saline-alkali degraded grasslands. In addition, higher salinity also affects water availability and soil oxygen concentration, limiting microbial respiration and growth (Hollister et al., 2010; Van Horn et al., 2014), which may explain the significant relationship between fungal communities and SWC.

### Effects of saline-alkali degradation on soil microbial community structure and composition in grassland

The difference of soil physical and chemical properties will also affect the composition of soil microbial community. The results of PCoA showed that saline-alkali degradation changed the composition of soil bacterial and fungal communities (**Figures 1C, D**). Wang et al. (2020) show that soil EC content, rather than soil pH, is the most important driver of microbial community composition. However, in this study, the results of random forest and Mantel tests showed that EC, pH and AP were the main drivers of soil bacterial community composition, while EC, pH and SOC were the main drivers of soil fungal community composition (**Figure 3**, **Table 2**). Our results were consistent with Rath and Rousk (2015). These results indicated that EC, pH, SOC and AP might be the soil factors to predict the composition of bacterial and fungal communities in saline-alkali soils. Soil salinity and pH can act as environmental filters for microbial communities and may lead to the enrichment or loss of certain species. Although salinized soils limit the growth and proliferation of most microorganisms, species with strong adaptations to low osmotic potential and high pH environments can survive and replace species with less resilient ones. Community composition of soil bacteria and fungi has been reported to depend on initial salinity, rather than final salinity (Rousk et al., 2011), which is inconsistent with our results. We found that differences in soil properties may lead to changes in microbial communities affected by salinization. There are unique OTUs in different gradients of saline-alkali degradation, and more salt-tolerant species replace less salt-tolerant species. For example, the unique bacterial OTUs in SD belonged to FCPU426 and CK-2C2-2 and the unique fungal OTUs in MD belonged to Calcarisporiellomycota (**Figures 1A, B**), which indicates that different soil salinity and pH have different filtration effects on soil bacteria and fungi. These phyla can adapt to the soil environment with high salinity and pH and can be used as indicator species of salin-alkali soil. Notably, we found that bacterial and fungal community composition was affected by soil nutrient content (SOC and AP). Studies have shown that exogenous nutrient input can also reduce the salinity pressure on microbial growth and diversity (Szoboszlay et al., 2019). Salt and alkali degradation changed the composition of plant community, the quantity and quality of plant litter and root exudates (Wu et al., 2014), and the increase of salt accelerated the leaching and erosion of soil organic matter. Changes in the survival substrate may drive changes in the number and composition of soil microbial communities (Langenheder et al., 2010; Yousuf et al., 2012). In addition, we found that the α diversity and unique OTUs of bacteria with different saline-alkali degradation gradients were significantly higher than those of fungi, which was also revealed by previous studies (Pankhurst et al., 2001; Sardinha et al., 2003), suggesting that fungi may be more sensitive to salinity than bacteria.

Actinobacteriota, Proteobacteria, Chloroflexi and Acidobacteria have been identified as the major bacterial communities in soil (Ramirez et al., 2014; Banerjee et al., 2018), which is consistent with this study. It has been reported that Actinobacteriota has the ability to decompose organic carbon and metabolize it at low temperatures (Yergeau et al., 2010). Most bacteria in the genus contain glutamic acid, glycine, alanine and meso-diaminophosphonic acid as cell wall binding amino acids (Bodenhausen et al., 2013; Niemho et al., 2013), it could be that they survive in a high salt environment. Proteobacteria is a fast-growing bacterium that is able to harness multiple carbon sources and light energy (Fierer et al., 2007; Mukhopadhya et al., 2012; Yao et al., 2017), bacteria with extensive degradation and habitat ability (Sinkko et al., 2013). Their salt tolerance ranges from pH 6.69 to 10.37 (Yang et al., 2019). They grow rapidly when the available substrate is unstable (e.g. artificially disturbed soil) (Zhang et al., 2016). Chloroflexi participated in photoautotrophic carbon fixation (Klatt et al., 2013), and its relative abundance was higher in saline soils than in non-saline soils (Mukhtar et al., 2016). Acidobacteria is related to the decomposition of organic matter and the balance of the microecological environment and is a major component of the microbial community in saline soil and Marine sediments (Ghosh et al., 2010; Mukhtar et al., 2016). Because of their diverse energy sources and strong salt tolerance, Actinobacteriota, Proteobacteria, Chloroflexi and Acidobacteria maintain high relative abundance under different saline-alkali degradation gradients. Ascomycota and Basidiomycota have been shown to be the dominant phylum of fungi in global soils (Tedersoo et al., 2014). Ascomycota plays an important role in the decomposition process and nutrient cycle of litters (Hartmann et al., 2017), and its nutritional modes include saprophytic, parasitic and symbiotic nutrition. Kim et al. (2019) showed that Ascomycota is highly salt-tolerant and has a high relative abundance in high salinity soils. Basidiomycota is a eutrophic type of bacteria that prefers resource-rich environments with high plant abundance (Sterkenburg et al., 2015). The lack of soil resources under saline-alkali degradation may be the reason why the relative abundance of Ascomycota and Basidiomycota did not change.

With the increase of salt-base degradation, the relative abundance of Actinobacteriota and Chytridiomycota showed a decreasing trend (**Figure 2**). The change of soil properties may be the driving cause of the relative abundance change of Actinobacteriota and Chytridiomycota. Mental test showed that the relative abundance of Actinobacteriota was significantly correlated with pH, SOC, TN, AP, SWC and soil cations (**Figure 5**). This was consistent with the results of Li et al. (2014) and Fierer et al. (2007). The relative abundance of Actinobacteriota is positively correlated with soil C and N content. SOC is the energy source of soil microorganisms (Cookson et al., 2008). Soil carbon and nitrogen contents are related, and soil microorganisms are directly or indirectly affected by influencing soil carbon to nitrogen ratio and pH value (Treseder, 2008; Serna-Chavez et al., 2013). Phosphorus can drive changes in the relative abundance of different bacteria by affecting soil pH (Jones et al., 2009). Therefore, the effect of saline-base degradation on the plant community and the change in soil properties induced by the change of plant community may be the cause of the relative abundance of Actinobacteriota. It is worth noting that Proteobacteria, Acidobacteriota, Ascomycota and Basidiomycota have no significant relationship with the physicochemical properties of salt-alkali soil, which means that these dominant phyla have certain regulating ability and tolerance to the filtration of soil salt and alkali.

## Conclusion

We studied the effects of different saline-alkali degradation gradients on soil microbial diversity and community composition in Songnen grassland. The degradation of grassland salt and alkali reduced the diversity of soil bacteria and fungi community and changed the composition of soil bacteria and fungi community. With the deterioration of salinity in grassland, the relative abundance of Actinobacteriota and Chytridiomycota decreased. EC, pH and AP were the main drivers of soil bacterial community composition, while EC, pH and SOC were the main drivers of soil fungal community composition. Different microorganisms are affected by different soil properties. The changes of plant community and soil environment are the main factors limiting the diversity and composition of soil microbial community. The results show that saline-alkali degradation of grassland has a negative effect on microbial biodiversity, so it is important to develop effective solutions to restore degraded grassland to maintain biodiversity and ecosystem function.

## Data availability statement

The datasets presented in this study can be found in online repositories. The names of the repository/repositories and accession number(s) can be found at: Sequence Read Archive (SRA) of the NCBI database under the BioProjectID PRJNA907217.

## Author contributions

ZB and MW conceived and designed this study. ZB drafted the original manuscript. MW provided very constructive suggestions for revisions. ZB, AJ, HL, MW and SQ contributed to the sampling and data analysis. All authors contributed to the article and approved the submitted version.
