# Supplementary material for: Explore the soil factors driving soil microbial community and structure in Songnen alkaline salt degraded grassland
Source: Front Plant Sci. 2023 May 9;14:1110685. doi: 10.3389/fpls.2023.1110685 (PMC10203596; doi:10.3389/fpls.2023.1110685)
Supplement: Supplementary file 1 [file DataSheet_1.doc]

Table S1. Vegetation of different degradation gradients. LD means the light degradation gradient , MD means the moderate degradation gradient and SD means the severe degradation gradient. Different letters (a, b, c) indicate significant effects.

| Degradation gradients | Coverage (%) | Height (cm) | Aboveground biomass (g/m^2^) | Number of substance | Main constructive species |
| --- | --- | --- | --- | --- | --- |
| LD | 84.67±5.69 a | 50.33±5.51 a | 234.88±18.91 a | 7.00±1.00 ab | *Leymus chinensis; Carex duriuscula* |
| MD | 63.33±0.41 b | 32.33±2.52 b | 163.23±13.72 b | 8.33±1.53 ab | *Leymus chinensis; Potentilla tanacetifolia* |
| SD | 33.33±5.69 c | 24.00±5.29 b | 99.25±18.06 c | 2.33±0.58 a | *Puccinellia tenuiflora; Chloris virgata* |

Table S2. Physical and chemical properties of soils with different degradation gradients. LD means the light degradation gradient , MD means the moderate degradation gradient and SD means the severe degradation gradient. Different letters (a, b, c) indicate significant effects. Abbreviations: soil water content (SWC); electrical conductivity (EC); soil organic carbon (SOC); total nitrogen (TN); available phosphorous(AP); Carbon nitrogen ratio (C/N)

| Degradation  gradients | SWC (%) | EC (mS/cm) | pH | SOC (g/kg) | TN (g/kg) | AP (mg/kg) | C/N | K^+^ (mg/kg) | Ca^2+^(mg/kg) | Mg^2+^ (mg/kg) | Na^+^ (mg/kg) | Exchangeable Na^+^ (cmol/kg) |
| --- | --- | --- | --- | --- | --- | --- | --- | --- | --- | --- | --- | --- |
| LD | 7.64±0.39a | 0.25±0.02b | 8.35±0.15c | 19.75±0.89a | 2.76±0.72a | 173.47±20.80a | 7.45±1.75a | 7.60±0.94b | 16.26±0.92c | 7.77±1.22b | 96.48±8.01c | 35.88±2.76b |
| MD | 5.92±0.63b | 0.38±0.03b | 9.01±0.28b | 15.89±0.36b | 1.71±0.31ab | 145.15±11.60a | 9.47±1.64a | 9.44±0.38b | 19.46±1.26b | 8.89±0.48b | 137.75±10.50b | 46.52±3.77a |
| SD | 4.53±0.55c | 1.29±0.36a | 10.20±0.03a | 6.83±0.79c | 6.83±0.79c | 97.10±12.63b | 12.15±3.89a | 13.32±1.14a | 24.12±1.12a | 11.01±0.63a | 176.89±8.86a | 52.30±3.43a |
