# Supplementary figures and images for: Explore the soil factors driving soil microbial community and structure in Songnen alkaline salt degraded grassland
Source: Front Plant Sci. 2023 May 9;14:1110685. doi: 10.3389/fpls.2023.1110685 (PMC10203596; doi:10.3389/fpls.2023.1110685)

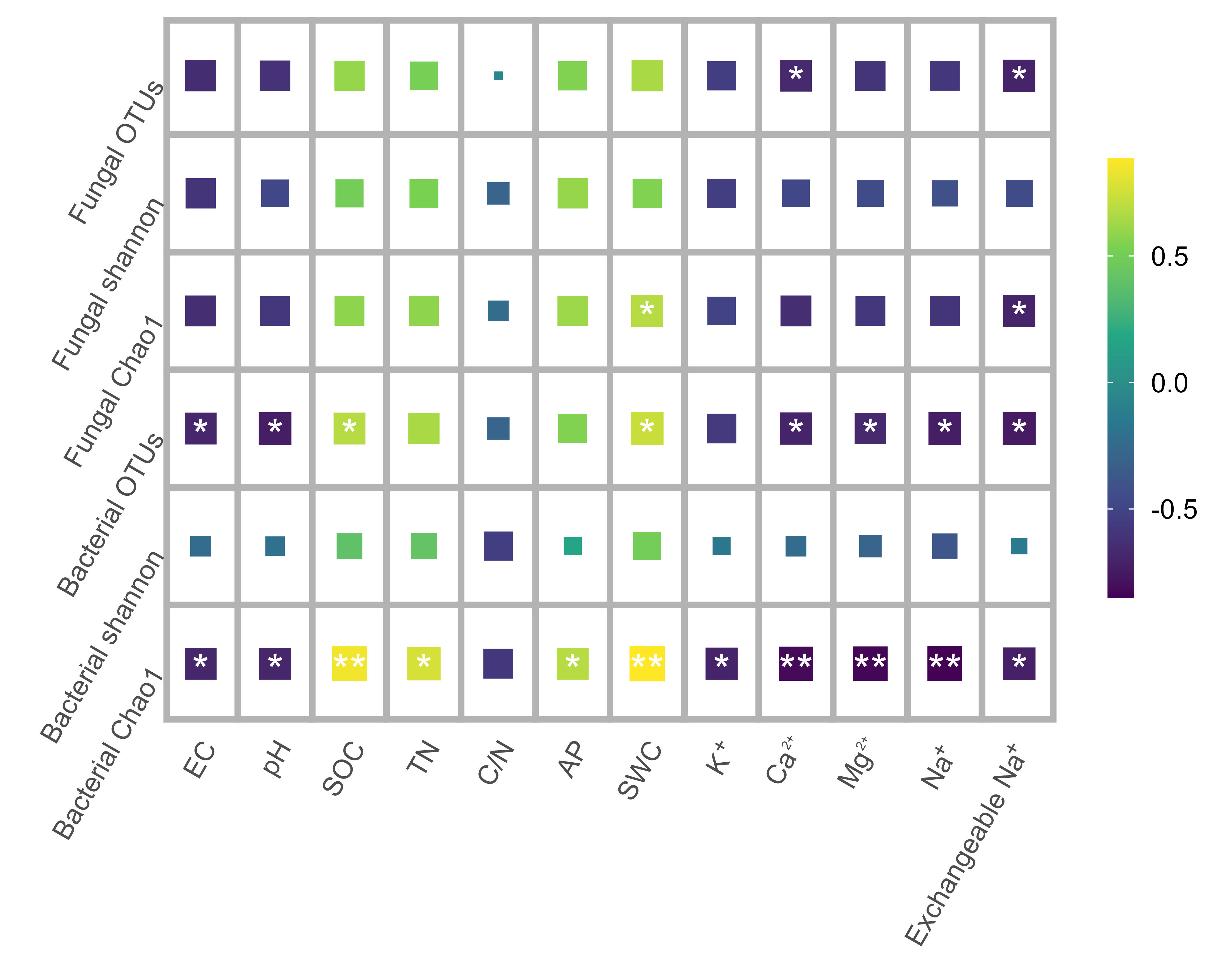

Supplement: Supplementary file 2 [file Image_1.jpeg]

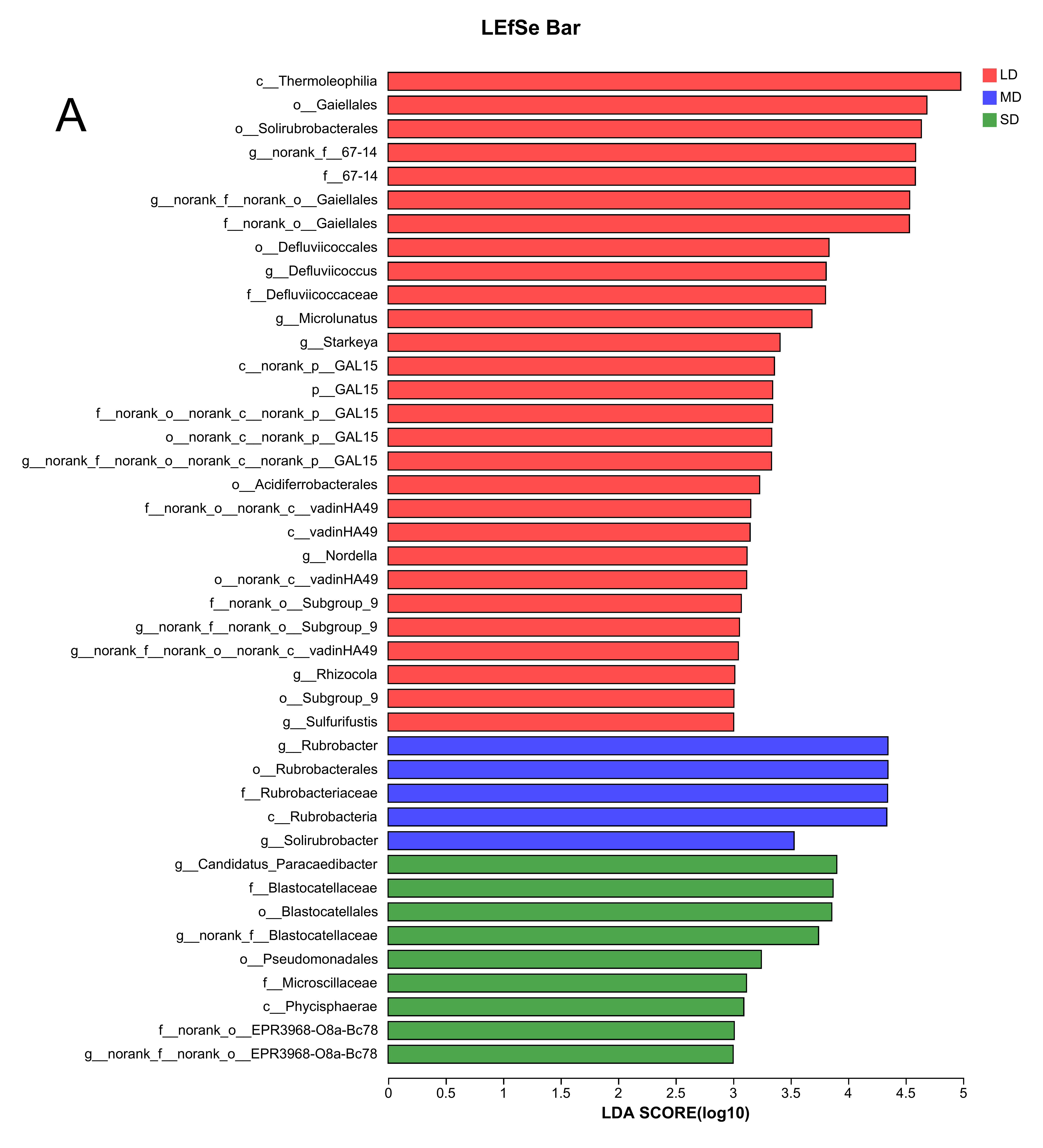

Supplement: Supplementary file 3 [file Image_2.jpeg]

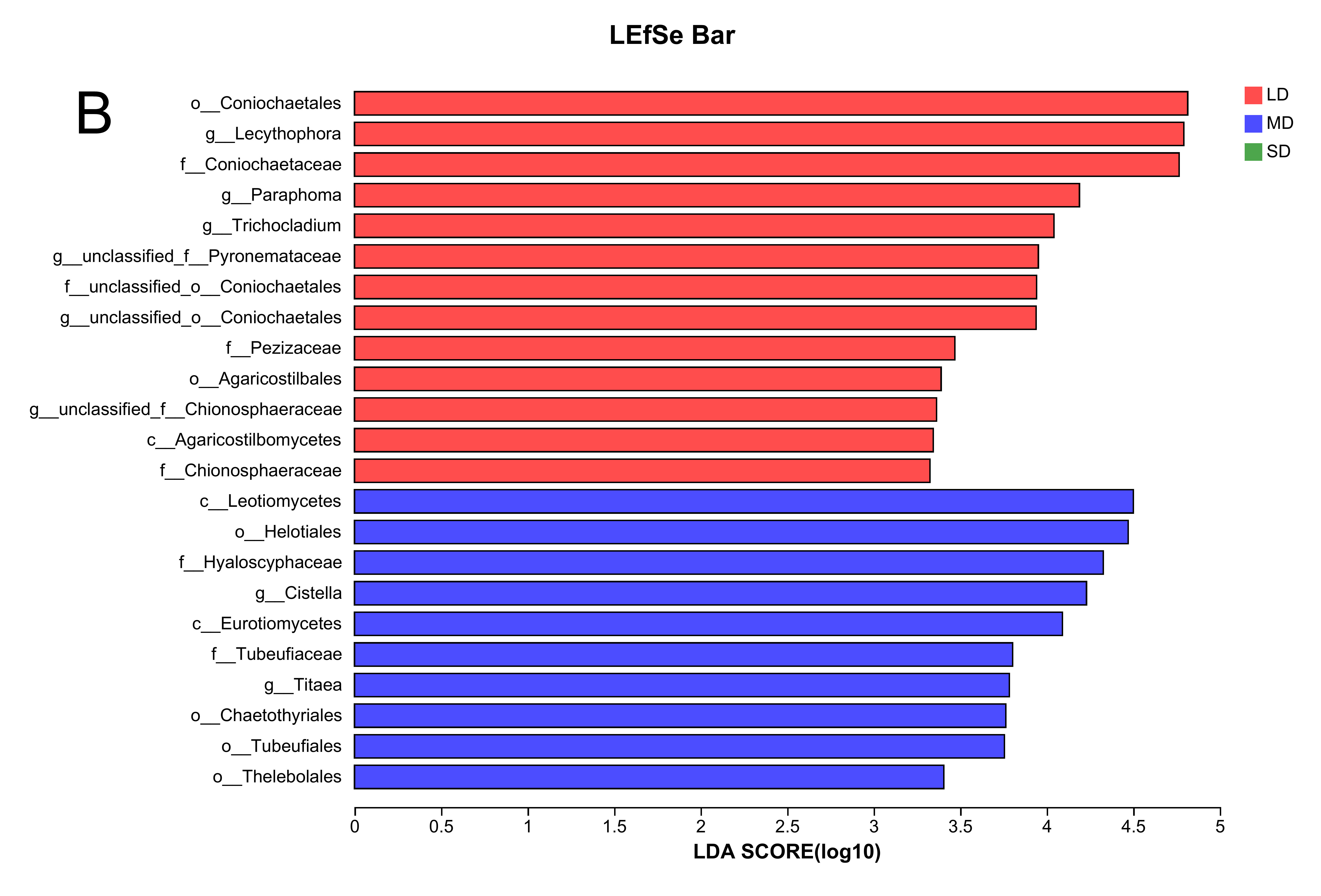

Supplement: Supplementary file 4 [file Image_3.jpeg]
